# DNA methylation biomarkers of myocardial infarction and cardiovascular disease

**DOI:** 10.1186/s13148-021-01078-6

**Published:** 2021-04-21

**Authors:** Alba Fernández-Sanlés, Sergi Sayols-Baixeras, Isaac Subirana, Mariano Sentí, S. Pérez-Fernández, Manuel de Castro Moura, Manel Esteller, Jaume Marrugat, Roberto Elosua

**Affiliations:** 1 Cardiovascular Epidemiology and Genetics Research Group, REGICOR Study Group, IMIM (Hospital del Mar Medical Research Institute), Dr Aiguader 88, 08003 Barcelona, Catalonia Spain; 2 Pompeu Fabra University (UPF), Barcelona, Catalonia Spain; 3 Medical Research Council (MRC) Integrative Epidemiology Unit, University of Bristol, Bristol, UK; 4 CIBER Cardiovascular Diseases (CIBERCV), Madrid, Spain; 5 Department of Medical Sciences, Molecular Epidemiology, Uppsala University, Uppsala, Sweden; 6 CIBER Epidemiology and Public Health (CIBERESP), Madrid, Spain; 7 Josep Carreras Leukaemia Research Institute (IJC), Badalona, Catalonia Spain; 8 CIBER Oncology (CIBERONC), Madrid, Spain; 9 Catalan Institution for Research and Advanced Studies (ICREA), Barcelona, Catalonia Spain; 10 Physiological Sciences Department, School of Medicine and Health Sciences, University of Barcelona (UB), Barcelona, Catalonia Spain; 11 Medicine Department, Faculty of Medicine, University of Vic-Central University of Catalonia (UVic-UCC), Vic, Catalonia Spain

**Keywords:** DNA methylation, Epigenome-wide association study, Predictive biomarkers, Myocardial infarction, Cardiovascular disease

## Abstract

**Background:**

The epigenetic landscape underlying cardiovascular disease (CVD) is not completely understood and the clinical value of the identified biomarkers is still limited. We aimed to identify differentially methylated loci associated with acute myocardial infarction (AMI) and assess their validity as predictive and causal biomarkers.

**Results:**

We designed a case–control, two-stage, epigenome-wide association study on AMI (n_discovery_ = 391, n_validation_ = 204). DNA methylation was assessed using the Infinium MethylationEPIC BeadChip. We performed a fixed-effects meta-analysis of the two samples. 34 CpGs were associated with AMI. Only 12 of them were available in two independent cohort studies (n ~ 1800 and n ~ 2500) with incident coronary and cardiovascular disease (CHD and CVD, respectively). The Infinium HumanMethylation450 BeadChip was used in those two studies. Four of the 12 CpGs were validated in association with incident CHD: *AHRR*-mapping cg05575921, *PTCD2-*mapping cg25769469, intergenic cg21566642 and *MPO*-mapping cg04988978. We then assessed whether methylation risk scores based on those CpGs improved the predictive capacity of the Framingham risk function, but they did not. Finally, we aimed to study the causality of those associations using a Mendelian randomization approach but only one of the CpGs had a genetic influence and therefore the results were not conclusive.

**Conclusions:**

We have identified 34 CpGs related to AMI. These loci highlight the relevance of smoking, lipid metabolism, and inflammation in the biological mechanisms related to AMI. Four were additionally associated with incident CHD and CVD but did not provide additional predictive information.

**Supplementary Information:**

The online version contains supplementary material available at 10.1186/s13148-021-01078-6.

## Introduction

Cardiovascular disease (CVD) and more specifically coronary heart disease (CHD) remains the number one cause of death and disease burden worldwide [1, 2]. At the individual level, prevention is based on the estimation of cardiovascular risk [3]. However, the sensitivity of cardiovascular risk estimation is low and a significant proportion of CHD events occurs in individuals classified as having moderate or low risk [4]. Additionally, the use of currently available drugs to control classical cardiovascular risk factors (CVRFs) does not prevent all CHD events, underlining the need to identify new strategies for reducing this residual cardiovascular risk [5]. Thus, information encoded in biological mechanisms should be unravelled to find new predictive biomarkers and potential therapeutic targets. Among these biomarkers, DNA methylation marks arise as emerging candidates.

DNA methylation is an epigenetic mechanism consisting on chemical modifications of cytosines, mostly followed by guanines (CpGs) [6]. Epigenome-wide association studies (EWASs) make it possible to find DNA methylation biomarkers of different traits and outcomes. In fact, DNA methylation pattern is associated with multiple chronic diseases [7], including CVD and CHD [8–13]. However, the clinical value of the identified biomarkers is still limited, and the epigenetic landscape underlying CVD is not completely understood.

The most common technology to assess DNA methylation is based on commercial arrays, which do not cover the whole methylome. Moreover, most current knowledge on the relation between DNA methylation and cardiovascular risk comes from studies based on the Infinium HumanMethylation450 BeadChip (Illumina, CA, USA; from now on, 450 k) [14] – which has been replaced by the Infinium MethylationEPIC BeadChip (Illumina, CA, USA; from now on, EPIC). Compared to the 450 k, EPIC interrogates 413,745 more methylation sites (but excludes 42,859) increasing the genomic coverage. Moreover, EPIC is enriched with functional sites analyses such as enhancers, DNase hypersensitive sites, and miRNA promoter regions [15]. Thus, the new chip has the potential to identify novel DNA methylation-based biomarkers of cardiovascular events.

We hypothesized that DNA methylation is associated with MI risk, and that some of these epigenetic marks could be predictive of future risk, and have causal effects on cardiovascular outcomes. Thus, this study had three aims: 1) to unravel genomic methylation loci associated with myocardial infarction (MI), 2) to assess their predictive capacity of cardiovascular risk, and 3) to decipher the causality of those associations.

## Results

### Quality control of DNA methylation data, cardiovascular outcomes and covariates

**Fig. 1:**
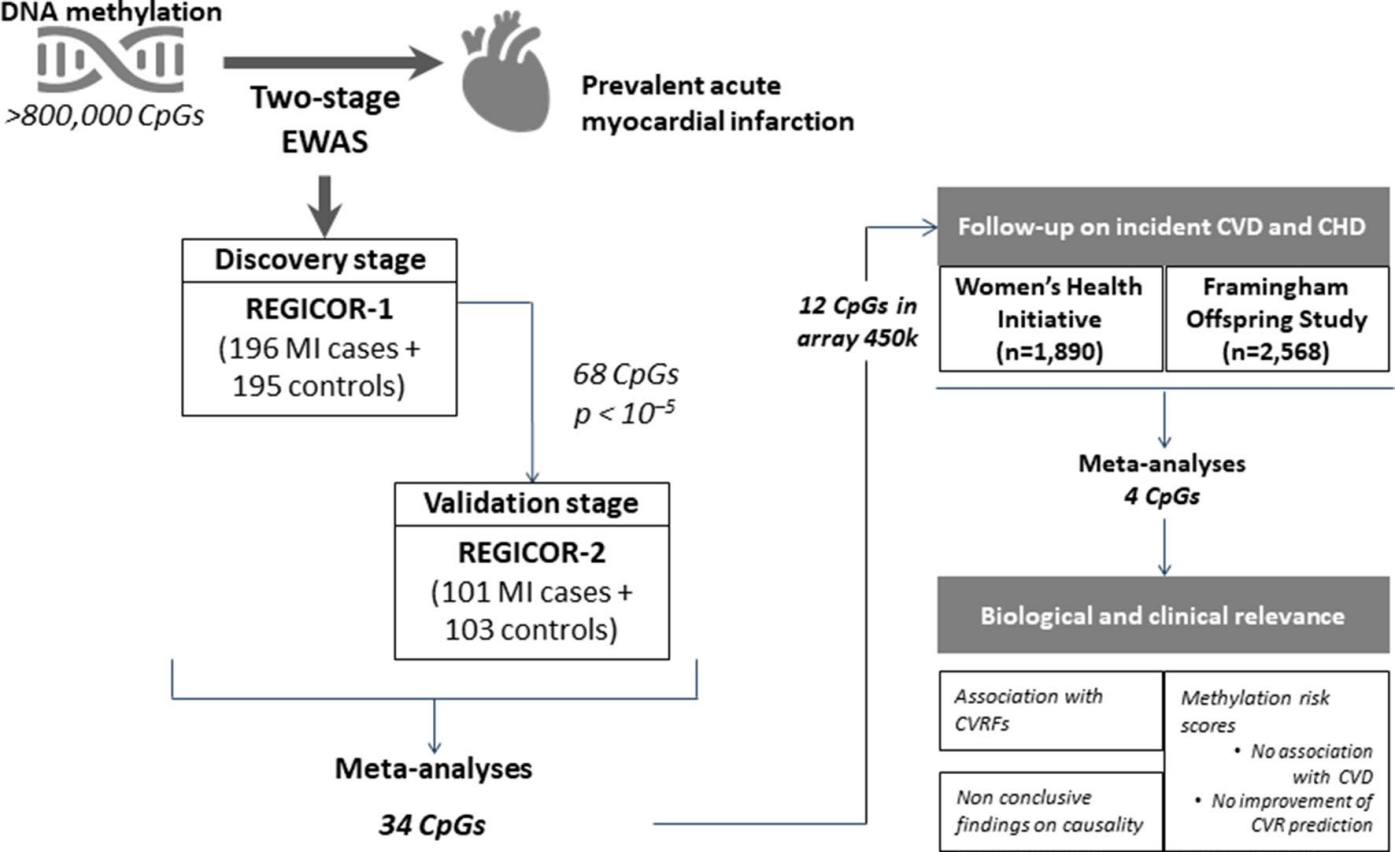
Flow chart of the steps included in this study

We finally included 391 individuals (196 cases and 195 controls) in the REGICOR-1 sample (Girona Heart Registry; REgistre GIroní del COR), 204 individuals (101 cases and 103 controls) in the REGICOR-2 sample, 1,863 women in the WHI Women’s Health Initiative) sample, and 2,540 participants in the FOS (Framingham Offspring Study) sample. The main sociodemographic and clinical characteristics of the three populations are shown in Tables 1 and 2. Regarding the number of CpGs, we analysed 811,610 CpGs in the REGICOR-1 sample, 820,183 CpGs in the REGICOR-2 sample, 478,369 CpGs in the WHI sample, and 483,656 CpGs in the FOS sample. Figure 1 illustrates the steps included in this study.

**Table 1:** Descriptive characteristics of the populations used in the two-stage EWAS on acute myocardial infarction (AMI): REGICOR-1 and REGICOR-2

Variables	Discovery: REGICOR-1		Validation: REGICOR-2	
	N = 391	NA‡	N = 204	NA‡
*AMI cases/controls, n (%)‡*				
AMI cases	196 (50.1)	0	101 (49.5)	0
Controls	195 (49.9)	0	103 (50.5)	0
*Age*	63.2 (6.94)	0	61.7 (6.90)	0
AMI cases	63.0 (6.96)	0	61.6 (6.83)	0
Controls	63.3 (6.94)	0	61.7 (7.01)	0
*Sex, female, n (%)*	201 (51.4)	0	100 (49.0)	0
AMI cases	100 (51.0)	0	49 (48.5)	0
Controls	101 (51.8)	0	51 (49.5)	0
*Smokers, n (%)*	93 (24.4)	10	67 (33.3)	3
AMI cases	76 (40.6)	9	45 (45.0)	1
Controls	17 (8.76)	1	22 (21.8)	2
*BMI, kg/m*^2^*‡	28.5 (4.75)	52	27.5 (4.87)	23
AMI cases	28.1 (4.34)	52	28.2 (5.87)	23
Controls	28.8 (5.02)	0	26.9 (3.90)	0
*Hypercholesterolaemia, n (%)‡*	179 (53.0)	53	93 (51.4)	23
AMI cases	93 (64.1)	51	51 (65.4)	23
Controls	86 (44.6)	2	42 (40.8)	0
*HTN, n (%)‡*	212 (57.1)	20	105 (54.1)	10
AMI cases	120 (68.2)	20	62 (68.1)	10
Controls	92 (47.2)	0	43 (41.7)	0
*Diabetes, n (%)*	87 (24.7)	39	44 (23.8)	19
AMI cases	56 (35.7)	39	27 (32.9)	19
Controls	31 (15.9)	0	17 (16.5)	0

^‡^NA, missing information; AMI, Acute myocardial infarction; BMI, Body mass index; Hypercholesterolaemia, defined as self-reported high cholesterol levels or treatment; HTN, Hypertension, defined as self-reported high blood pressure or treatment; Diabetes, defined as self-reported diabetes or treatment

^†^Median (Interquartile Range)

*Mean (Standard deviation)

**Table 2:** Descriptive characteristics of the populations used in the follow-up association studies on incident cardiovascular (CVD) and coronary heart disease (CHD) events: Women’s Health Initiative (WHI) and Framingham Offspring Study (FOS)

Variables	WHI		FOS CHD		FOS CVD	
	N = 1863	NA	N = 2266	NA	N = 2123	NA
Age	64.2 (7.03)	0	65.7 (8.82)	0	65.3 (8.66)	0
Sex, female, n (%)	1863 (100)	0	1284 (56.7)	0	1206 (56.8)	0
CHD, n (%)^‡^	914 (49.1)	0	106 (4.68)	0	_	_
CVD, n (%)^‡^	984 (52.8)	0	_	_	222 (10.5)	0
Smokers, n (%)	174 (9.46)	24	217 (9.62)	11	202 (9.56)	11
BMI, kg/m2*^‡^	29.8 (5.97)	11	28.1 (5.39)	8	28.0 (5.33)	6
Total cholesterol, mg/dl*	233 (42.3)	1	189 (36.2)	1	190 (35.8)	1
LDL-C, mg/dl*^‡^	152 (38.7)	31	107 (30.8)	2	108 (30.5)	2
HDL-C, mg/dl*^‡^	52.2 (13.2)	1	58.3 (18.3)	2	58.7 (18.3)	2
TG, mg/d^†^	126 [93.0;176]	1	101 [73.0;140]	1	100 [73.0;139]	1
SBP, mmHg*^‡^	131 (17.5)	0	126 (16.9)	1	125 (16.7)	1
DBP, mmHg*^‡^	76.4 (9.27)	0	72.1 (9.99)	3	72.3 (9.92)	3
HTN treatment, n (%)^‡^	672 (45.9)	400	1025 (45.4)	7	927 (43.8)	6
Glucose, mg/dl^†^	107 (38.3)	1	106 (21.8)	2	105 (21.5)	2
Diabetes, n (%)	319 (17.1)	0	307 (14.3)	126	266 (13.3)	119

^‡^CHD, Coronary heart disease; CVD, Cardiovascular disease; BMI, Body mass index; LDL-C, Cholesterol in low-density lipoprotein; HDL-C, Cholesterol in high-density lipoprotein; TG, Triglycerides; SBP, Systolic blood pressure; DBP, Diastolic blood pressure; HTN: hypertension; Diabetes, defined as previous treatment or glycaemia ≥ 126 mg/dl

^†^Median (Interquartile range)

*Mean (Standard deviation)

### Association between DNA methylation and cardiovascular outcomes

#### Two-stage EWAS on acute myocardial infarction

##### Discovery stage

The associations from the discovery stage (REGICOR-1) that were taken to the subsequent validation (*p-value* < 10^–5^), and their Manhattan and Q-Q plots are shown in the Additional file 2: Table S1, and Additional file 1: Figs. S1 and S2]. In total, we identified 68 CpGs suggestively related to MI (Additional file 1: Fig. S3). Model 1 provided 56 CpGs, of which three were also found in both model 2 and 3, and 13 in model 2. One additional CpG was found in both model 2 and 3, two in model 2 and nine in model 3.

##### Validation and meta-analysis

The association studies performed in the validation stage included the 68 CpGs suggestively related to MI. We meta-analysed the results of those 68 associations from both stages. We identified 34 differentially methylated CpGs related to MI, with similar effect sizes in all three models for most of the CpGs (except cg21566642, cg05575921, cg03636183). The 34 CpGs were located in 25 different loci (26 genes, with one CpG mapping to two genes) and nine intergenic regions (Table 3, and Additional file 2: Table S2).

**Table 3:** CpGs differentially methylated in association with prevalent myocardial infarction in the fixed-effects meta-analyses of the REGICOR case–control samples

Genomic information				Model 1			Model 2			Model 3		
CpG	Chr*	Position (bp)	Gene	Coef*	SE*	*P*	Coef*	SE*	*P*	Coef*	SE*	*P*
cg15724772	1	6,373,534	*ACOT7*	1.17	1.94 × 10^–1^	1.76 × 10^–9^	1.16	2.17 × 10^–1^	8.14 × 10^–8^	1.00	2.30 × 10^–1^	1.38 × 10^–5^
cg11919692	1	26,343,868	Intergenic	1.08	1.98 × 10^–1^	4.94 × 10^–8^	1.11	2.19 × 10^–1^	4.00 × 10^–7^	1.04	2.34 × 10^–1^	9.29 × 10^–6^
cg10636246	1	159,046,973	*AIM2*	− 0.96	1.41 × 10^–1^	1.03 × 10^–11^	− 0.98	1.58 × 10^–1^	5.21 × 10^–10^	− 0.86	1.68 × 10^–1^	3.68 × 10^–7^
cg08345526	1	226,916,320	*ITPKB*	1.79	2.93 × 10^–1^	1.02 × 10^–9^	1.85	3.29 × 10^–1^	1.71 × 10^–8^	1.60	3.45 × 10^–1^	3.42 × 10^–6^
cg01552966	2	25,528,330	*DNMT3A*	1.15	1.93 × 10^–1^	2.43 × 10^–9^	1.25	2.22 × 10^–1^	1.78 × 10^–8^	1.04	2.33 × 10^–1^	7.66 × 10^–6^
cg25420477	2	70,319,121	Intergenic	1.54	2.52 × 10^–1^	1.08 × 10^–9^	1.63	2.84 × 10^–1^	9.01 × 10^–9^	1.47	3.01 × 10^–1^	9.45 × 10^–7^
cg06528771	2	102,513,985	Intergenic	− 1.71	2.79 × 10^–1^	9.88 × 10^–10^	− 1.89	3.16 × 10^–1^	2.26 × 10^–9^	− 1.81	3.48 × 10^–1^	1.81 × 10^–7^
cg12206840	2	136,826,451	Intergenic	1.12	1.98 × 10^–1^	1.31 × 10^–8^	1.12	2.23 × 10^–1^	4.89 × 10^–7^	1.03	2.33 × 10^–1^	9.08 × 10^–6^
cg21566642	2	233,284,661	Intergenic	− 0.65	1.13 × 10^–1^	7.70 × 10^–9^	− 0.30	1.56 × 10^–1^	5.26 × 10^–2^	− 0.41	1.70 × 10^–1^	1.50 × 10^–2^
cg10650214	3	45,914,188	*LZTFL1*	1.28	1.97 × 10^–1^	8.01 × 10^–11^	1.26	2.19 × 10^–1^	8.05 × 10^–9^	1.18	2.31 × 10^–1^	2.99 × 10^–7^
cg07386061	3	52,492,874	*NISCH*	0.99	1.79 × 10^–1^	2.86 × 10^–8^	1.03	2.01 × 10^–1^	3.27 × 10^–7^	0.86	2.09 × 10^–1^	4.01 × 10^–5^
cg13373048	3	71,892,575	Intergenic	− 2.76	3.57 × 10^–1^	1.02 × 10^–14^	− 3.24	4.23 × 10^–1^	1.93 × 10^–14^	− 3.03	4.29 × 10^–1^	1.77 × 10^–12^
cg03733470	3	196,613,114	*SENP5*	− 1.20	2.07 × 10^–1^	5.94 × 10^–9^	− 1.28	2.30 × 10^–1^	2.70 × 10^–8^	− 1.17	2.46 × 10^–1^	2.01 × 10^–6^
cg05575921	5	373,378	*AHRR*	− 0.75	1.25 × 10^–1^	2.05 × 10^–9^	− 0.40	1.91 × 10–1	3.48 × 10^–2^	− 0.45	2.04 × 10^–1^	2.87 × 10^–2^
cg25769469	5	71,643,841	*PTCD2*	− 1.33	2.20 × 10^–1^	1.33 × 10^–9^	− 1.38	2.53 × 10^–1^	5.40 × 10^–8^	− 1.41	2.80 × 10^–1^	4.92 × 10^–7^
cg04725636	8	67,004,538	*DNAJC5B*	− 0.80	1.45 × 10^–1^	3.33 × 10^–8^	− 0.72	1.59 × 10^–1^	5.47 × 10^–6^	− 0.75^1^	1.74 × 10^–1^	1.76 × 10^–5^
cg12066594	9	127,162,066	*PSMB7*	− 1.89	3.37 × 10^–1^	2.00 × 10^–8^	− 2.57	4.07 × 10^–1^	2.67 × 10^–10^	− 2.36	4.25 × 10^–1^	2.83 × 10^–8^
cg24501073	11	34,210,305	*ABTB2*	0.99	1.73 × 10^–1^	1.07 × 10^–8^	0.98	1.96 × 10^–1^	6.11 × 10^–7^	0.82	1.98 × 10^–1^	3.07 × 10^–5^
cg02042026	11	113,936,782	*ZBTB16*	1.04	1.70 × 10^–1^	8.50 × 10^–10^	0.99	1.88 × 10^–1^	1.51 × 10^–7^	0.98	2.04 × 10^–1^	1.50 × 10^–6^
cg01381203	12	2,985,630	*FOXM1;RHNO1*	− 0.88	1.62 × 10^–1^	5.17 × 10^–8^	− 0.84	1.76 × 10^–1^	1.88 × 10^–6^	− 0.92	1.95 × 10^–1^	2.44 × 10^–6^
cg02175213	12	69,352,407	*CPM*	− 1.08	1.85 × 10^–1^	5.15 × 10^–9^	− 1.10	2.06 × 10^–1^	9.76 × 10^–8^	− 0.82	2.14 × 10^–1^	1.27 × 10^–4^
cg06084585	13	50,797,125	*DLEU1*	− 1.60	2.87 × 10^–1^	2.37 × 10^–8^	− 1.47	3.14 × 10^–1^	2.96 × 10^–6^	− 1.36	3.30 × 10^–1^	3.54 × 10^–5^
cg15867698	14	69,438,267	*ACTN1*	1.13	1.86 × 10^–1^	1.58 × 10^–9^	1.26	2.14 × 10^–1^	3.33 × 10^–9^	1.14	2.25 × 10^–1^	4.04 × 10^–7^
cg07252680	14	94,857,224	*SERPINA1*	− 1.56	2.41 × 10^–1^	1.17 × 10^–10^	− 1.43	2.65 × 10^–1^	6.57 × 10^–8^	− 1.41	2.86 × 10^–1^	7.93 × 10^–7^
cg16125725	15	70,101,302	Intergenic	− 2.03	3.45 × 10^–1^	4.02 × 10^–9^	− 2.30	3.97 × 10^–1^	6.82 × 10^–9^	− 2.20	4.18 × 10^–1^	1.39 × 10^–7^
cg09967176	15	77,280,925	Intergenic	− 1.28	1.78 × 10^–1^	6.93 × 10^–13^	− 1.40	2.03 × 10^–1^	5.21 × 10^–12^	− 1.16	2.10 × 10^–1^	3.82 × 10^–8^
cg17261469	16	87,420,879	*FBXO31*	0.99	1.73 × 10^–1^	9.49 × 10^–9^	1.00	1.94 × 10^–1^	3.01 × 10^–7^	1.00	2.04 × 10^–1^	9.92 × 10^–7^
cg04983687	16	88,558,223	*ZFPM1*	0.72	1.29 × 10^–1^	2.61 × 10^–8^	0.68	1.42 × 10^–1^	1.79 × 10^–6^	0.44	1.46 × 10^–1^	2.49 × 10^–3^
cg22508829	17	17,843,585	*TOM1L2*	1.28	1.96 × 10^–1^	7.97 × 10^–11^	1.35	2.26 × 10^–1^	2.41 × 10^–9^	1.10	2.28 × 10^–1^	1.23 × 10^–6^
cg22146772	17	26,793,673	Intergenic	1.23	1.97 × 10^–1^	5.38 × 10^–10^	1.18	2.22 × 10^–1^	8.98 × 10^–8^	1.06	2.28 × 10^–1^	3.31 × 10^–6^
cg13064873	17	48,360,726	*TMEM92*− *AS1*	− 1.08	1.85 × 10^–1^	5.31 × 10^–9^	− 1.19	2.12 × 10^–1^	1.82 × 10^–8^	− 1.04	2.23 × 10^–1^	3.19 × 10^–6^
cg04988978	17	56,359,578	*MPO*	− 1.31	2.07 × 10^–1^	2.33 × 10^–10^	− 1.45	2.37 × 10^–1^	9.71 × 10^–10^	− 1.22	2.43 × 10^–1^	5.41 × 10^–7^
cg03636183	19	17,000,585	*F2RL3*	− 0.74	1.36 × 10^–1^	4.56 × 10^–8^	− 0.35	1.80 × 10^–1^	5.24 × 10^–2^	− 0.39	1.90 × 10^–1^	4.12 × 10^–2^
cg19448292	20	35,504,064	*C20orf118*	1.24	1.93 × 10^–1^	1.17 × 10^–10^	1.38	2.25 × 10^–1^	7.89 × 10^–10^	1.15	2.30 × 10^–1^	6.05 × 10^–7^

*Chr, Chromosome; Coef; Coefficient of association; SE, Standard error

Model 1 was adjusted for estimated cell counts and two surrogate variables. Model 2 was further adjusted for smoking status. Model 3 was additionally adjusted for diabetes, hypercholesterolemia, and hypertension. The Bonferroni-corrected *p* threshold was 6.17 × 10^–8^

#### Follow-up association studies on incident CHD and CVD events

Out of the 34 identified CpGs associated with MI, only 12 were available in the samples with incident cases (whose DNA methylation was profiled with the array 450 k). In total, we validated four CpGs after the meta-analysis of the separate association studies in the WHI and the FOS samples (*p-value* < 0.05/12 = 4.17 × 10^–3^): *AHRR*-mapping cg05575921, *PTCD2-*mapping cg25769469, intergenic cg21566642 and *MPO*-mapping cg04988978. The four CpGs were associated with CHD but cg25769469 was not related to CVD (Table 4, Additional file 2: Table S3).

**Table 4:** CpGs differentially methylated in association with incident coronary/cardiovascular disease in the fixed-effects meta-analyses of the samples from the Women’s Health Initiative (WHI) and the Framingham Offspring Study (FOS)

Genomic information				Model 1			Model 2			Model 3		
CpG	Chr*	Position (bp)	Gene	Coef*	SE*	*P*	Coef*	SE*	*P*	Coef*	SE*	*P*
*Incident coronary heart disease*												
cg21566642	2	233,284,661	Intergenic	− 1.34 × 10^–1^	4.33 × 10^–2^	2.00 × 10^–3^	− 9.63 × 10^–2^	4.83 × 10^–2^	4.62 × 10^–2^	− 9.66 × 10^–2^	5.39 × 10^–2^	7.32 × 10^–2^
cg05575921	5	373,378	*AHRR*	− 1.80 × 10^–1^	4.73 × 10^–2^	1.38 × 10^–4^	− 1.33 × 10^–1^	5.81 × 10^–2^	2.21 × 10^–2^	− 1.22 × 10^–1^	6.52 × 10^–2^	6.05 × 10^–2^
cg25769469	5	71,643,841	*PTCD2*	− 2.62 × 10^–1^	7.94 × 10^–2^	9.67 × 10^–4^	− 2.52 × 10^–1^	8.02 × 10^–2^	1.70 × 10^–3^	− 2.14 × 10^–1^	8.89 × 10^–2^	1.59 × 10^–2^
cg04988978	17	56,359,578	*MPO*	− 2.28 × 10^–1^	7.49 × 10^–2^	2.29 × 10^–3^	− 2.07 × 10^–1^	7.58 × 10^–2^	6.19 × 10^–3^	− 1.06 × 10^–1^	8.35 × 10^–2^	2.03 × 10^–1^
Incident cardiovascular disease												
cg21566642	2	233,284,661	Intergenic	− 1.68 × 10^–1^	3.97 × 10^–2^	2.34 × 10^–5^	− 1.31 × 10^–1^	4.40 × 10^–2^	2.94 × 10^–3^	− 1.37 × 10^–1^	4.85 × 10^–2^	4.87 × 10^–3^
cg05575921	5	373,378	*AHRR*	− 2.08 × 10^–1^	4.30 × 10^–2^	1.30 × 10^–6^	− 1.67 × 10^–1^	5.24 × 10^–2^	1.42 × 10^–3^	− 1.61 × 10^–1^	5.79 × 10^–2^	5.54 × 10^–3^
cg25769469	5	71,643,841	*PTCD2*	− 1.92 × 10^–1^	7.27 × 10^–2^	8.40 × 10^–3^	− 1.77 × 10^–1^	7.35 × 10^–2^	1.58 × 10^–2^	− 1.26 × 10^–1^	8.09 × 10^–2^	1.20 × 10^–1^
cg04988978	17	56,359,578	*MPO*	− 2.04 × 10^–1^	6.72 × 10^–2^	2.42 × 10^–3^	− 1.83 × 10^–1^	6.79 × 10^–2^	6.99 × 10^–3^	− 9.08 × 10^–2^	7.36 × 10^–2^	2.18 × 10^–1^

*Chr, Chromosome; Coef; Coefficient of association; SE, Standard error

Model 1 was adjusted for age, estimated cell counts and two surrogate variables (plus ancestry in WHI, plus sex in FOS). Model 2 was further adjusted for smoking status. Model 3 was additionally adjusted for diabetes, hypercholesterolemia, and hypertension. The Bonferroni-corrected *p* threshold was 4.17 × 10^–3^

### Association between the identified CpGs and CVRFs

Table 5 shows the associations observed between the identified CpGs and classical CVRFs. The four validated CpGs were related to some CVRF [*p-value* < 0.05/(4 CpGs*8 CVRF) = 1.56 × 10^–3^].

**Table 5:** Associations between the identified CpGs and classical cardiovascular risk factors (CVRFs) in the fixed-effects meta-analyses of the four samples

CpG	Smoking	BMI*	HDL-C*	LDL-C*	TG*	Glucose	SBP	DBP*	
cg21566642	− 1.61	9.55 × 10^–3^	7.69 × 10^–4^	− 1.10 × 10^–3^	− 4.17 × 10^–4^	2.32 × 10^–4^	2.30 × 10^–3^	7.97 × 10^–3^	Coefficient
	4.22 × 10^–2^	2.65 × 10^–3^	1.21 × 10^–3^	4.09 × 10^–4^	1.95 × 10^–4^	4.20 × 10^–4^	9.02 × 10^–4^	1.67 × 10^–3^	SE^*^
	< 2.2 × 10^–16^	3.21 × 10^–4^	5.24 × 10^–1^	7.11 × 10^–3^	3.22 × 10^–2^	5.81 × 10^–1^	1.08 × 10^–2^	1.78 × 10^–6^	*P*
cg05575921	− 1.61	1.17 × 10^–2^	4.36 × 10^–3^	− 1.35 × 10^–3^	− 7.09 × 10^–4^	1.86 × 10^–4^	2.15 × 10^–3^	7.54 × 10^–3^	Coefficient
	4.22 × 10^–2^	2.46 × 10^–3^	1.11 × 10^–3^	3.77 × 10^–4^	1.79 × 10^–4^	3.87 × 10^–4^	8.23 × 10^–4^	1.52 × 10^–3^	SE^*^
	< 2.2 × 10^–16^	2.22 × 10^–6^	8.22 × 10^–5^	3.39 × 10^–4^	7.63 × 10^–5^	6.30 × 10^–1^	9.06 × 10^–3^	7.69 × 10^–7^	*P*
cg25769469	− 9.01 × 10^–2^	− 1.12 × 10^–2^	6.76 × 10^–3^	2.47 × 10^–4^	− 1.38 × 10^–3^	− 1.52 × 10^–3^	− 2.35 × 10^–3^	− 4.17 × 10^–3^	Coefficient
	5.08 × 10^–2^	2.64 × 10^–3^	1.20 × 10^–3^	4.11 × 10^–4^	1.93 × 10^–4^	4.17 × 10^–4^	9.10 × 10^–4^	1.69 × 10^–3^	SE^*^
	7.65 × 10^–2^	2.32 × 10^–5^	1.92 × 10^–8^	5.48 × 10^–1^	8.75 × 10^–13^	2.69 × 10^–4^	9.97 × 10^–3^	1.36 × 10^–2^	*P*
cg04988978	− 7.29 × 10^–2^	− 1.15 × 10^–2^	5.28 × 10^–3^	3.59 × 10^–4^	− 1.19 × 10^–3^	− 1.88 × 10^–3^	− 2.88 × 10^–3^	− 3.54 × 10^–3^	Coefficient
	5.15 × 10^–2^	2.64 × 10^–3^	1.20 × 10^–3^	4.08 × 10^–4^	1.92 × 10^–4^	4.15 × 10^–4^	9.02 × 10^–4^	1.67 × 10^–3^	SE^*^
	1.57 × 10^–1^	1.26 × 10^–5^	1.08 × 10^–5^	3.80 × 10^–1^	5.79 × 10^–10^	5.97 × 10^–6^	1.41 × 10^–3^	3.47 × 10^–2^	*P*

*BMI, Body mass index; HDL-C, Cholesterol in high-density lipoprotein; LDL-C, Cholesterol in low-density lipoprotein; TG, Triglycerides; SBP, Systolic blood pressure; DBP, Diastolic blood pressure; SE, Standard Error

### Association between MRSs and incidence of CHD and CVD

The associations between the methylation risk scores (MRSs) and the incidence of coronary (n = 94) and cardiovascular (n = 222) events in the FOS population are shown in Additional file 2: Table S4. The median of the follow-up periods for CVD and CHD incidence were 7.67 and 7.87 years, respectively. The MRSs were not associated with higher cardiovascular risk independently of the classical CVRFs. Consistently, the addition of any of the MRSs to the Framingham risk function did not improve its predictive capacity in the FOS cohort (Additional file 2: Table S4).

### Causality of the associations between DNA methylation and cardiovascular outcomes

Of the four identified CpGs, only cg21566642 showed a genetic influence; its methylation levels in adolescence were associated with rs72617176 and in childhood with rs139595493. We did not have individual data to test the first and second Mendelian randomization assumptions, but the meQTLs were associated with the CpGs methylation levels at genome-wide significance independently of age, sex or ancestry principal components [16]. Only the Wald ratio method could be conducted, since it uses a single instrumental variable. The results did not support a causal effect of methylation at cg21566642 on either MI or CHD (Additional file 2: Table S5). We could not perform sensitivity tests for pleiotropic effects or its strength. The other three CpGs could not be instrumented.

## Discussion

We have identified 34 methylation loci associated with acute MI in a two-stage EWAS, analysing ~ 850,000 CpGs. All but two of these MI-associated sites (cg05575921 located in *AHRR* and the intergenic cg21566642) are newly reported. Of those, 12 CpGs could be studied in association with incident cases of CHD and CVD, and we identified four of them associated with incident CHD (three of them also with incident CVD). All four were also related to traditional CVRFs, supporting their role in the development of these diseases. However, their clinical utility as predictive biomarkers or drug targets was not proven.

Recently, two EWASs on incident CHD were published providing different findings from ours. Ward-Caviness et al.found nine CpGs associated with incident acute MI [9]. Agha et al. reported 52 CpGs related to incident CHD [8]. None of them was replicated in our study. This lack of concordance could be related to methodological differences (incident vs prevalent cases; myocardial infarction vs CHD; considered confounder variables; characteristics of the populations), and highlights the complexity of the study of these diseases.

### CpG sites associated with acute MI events

The 34 identified CpGs showed similar effect sizes in the two REGICOR samples and we considered them potentially relevant. Similarly, all but three CpGs (*AHRR*-mapping cg05575921, *F2RL3*-mapping cg03636183, and the intergenic cg21566642) showed consistent effect sizes in the three models. The effect size of those three was reduced by half when adjusted for smoking, which highlights the important role of this risk factor in the MI context. In fact, all three sites are widely described to be related to smoking [17–19].

Differentially methylated genes were enriched in diverse molecular and physiological pathways, including lipid metabolism and metabolic and inflammatory diseases, underlining their relevance on the pathogenesis of CHD. Interestingly, the *SERPINA1* locus also anchors genetic variants related to CHD [20], and other identified loci present with genetic variants associated with body mass index (*DNMT3A*, *ABTB2*, *ZBTB16*, *NISCH*, *AHRR*, *DLEU1*), inflammatory biomarkers or blood cell counts (*AIM2*, *ITPKB*, *DNMT3A*, *LZTFL1*, *PSMB7*, *ZBTB16*, *ACTN1*, *SERPINA1*, *MPO, DNAJC5B, CPM*, *DLEU1, ZFPM1*), blood pressure (*PTCD2, PSMB7, SERPINA1*, *AHRR*) and lipids (*SERPINA1, NISCH*, *DLEU1, ZFPM1*) [21].

Nonetheless, the case–control design of our initial discovery sample limits the inference of the biological sequence of the epigenetic marks, the related biological mechanisms, and the clinical event. One possible scenario could be that the identified DNA methylation marks occurred before the acute event, as potential biological mechanisms involved in MI pathogenesis. This may be the case of the three CpGs that were related to smoking. Conversely, as blood samples of MI cases were collected within the initial 24 h after hospitalization, the other possibility could be that methylation at the identified CpGs had changed as a consequence of the acute event or the therapeutic procedures. If the first scenario can be proven in further studies, these DNA methylation marks could be potential predictive biomarkers of MI or new therapeutic targets. If they are found to be post-MI marks, further studies could evaluate their potential as biomarkers of prognosis.

### CpG sites consistently related to prevalent and incident CVD events

Twelve of the 34 identified CpGs could be evaluated in prospective samples and four of them were also related to incident cases of CHD. cg21566642 maps to an intergenic region, and cg05575921, cg04988978 and cg25769469 annotate to *AHRR, MPO* and *PTCD2*, respectively. To our knowledge, these CpGs were not associated with cardiovascular events in previous EWAS reports.

cg21566642 and cg05575921 were highly and inversely associated with smoking, which is supported by previous EWAS [18, 19]. We have also previously reported both CpGs as related to age-independent cardiovascular risk [13], and they have been related to all-cause mortality in an EWAS [22]. cg05575921 was further associated directly with cholesterol in high-density lipoproteins (HDL-C) and inversely with cholesterol in low-density lipoproteins (LDL-C) and triglyceride levels in our study. This CpG has been related to both CHD prevalence and incidence in a candidate gene study [23].

cg04988978 and cg25769469 annotate to *MPO* and *PTCD2*, respectively. Both CpGs were associated directly with HDL-C and inversely with triglyceride and glucose levels. MPO encodes the myeloperoxidase, which promotes atherosclerotic lesions by enhancing APOB oxidation within low-density lipoproteins [24] and was causally associated with incident cardiovascular outcomes [25]. One CpG located within PTCD2 was previously identified to be associated with hypertension in obstructive sleep apnea patients [26], and genetic variants in this gene have been related with blood pressure [21].

### MRSs as predictive CVD biomarkers

To assess the value of the four identified CpGs as predictive biomarkers, we followed the AHA recommendations [27]. However, neither we observed an independent association between the MRSs and the incidence of CVD events in the FOS, nor we observed an improvement in the predictive capacity of the Framingham risk function when including this score. This highlights the challenge of novel biomarkers to improve cardiovascular risk prediction.

### Causality of the associations between methylation loci and cardiovascular outcomes

The four CpGs associated not only with acute MI, but also incident CHD, may suggest that DNA methylation changes at those loci occur prior to the event. However, this association does not guarantee whether differential DNA methylation at those loci has a causal effect on CHD. Mendelian randomization can be used to ascertain this causal relationship. However, this approach could only be undertaken for cg21566642. Although a non-causal relationship was suggested, this must be interpreted with caution as there was a single genetic instrumental variable, and we cannot discard that the meQTL is in linkage disequilibrium with the causal variant for CHD, reverse causation or horizontal pleiotropy using this framework [28, 29]. Moreover, cg21566642 showed a genetic influence in childhood and adolescence, while CHD events typically occur during adulthood.

### Strengths and limitations

The main strength of our study is that it is the first two-stage EWAS on MI to be based on more than 800,000 CpGs across the genome. Moreover, we aimed to validate our findings in prospective samples of CHD and CVD as a proxy of MI. Also, we aimed to prove the clinical relevance of our findings. However, some limitations should be acknowledged. First, two thirds of the CpGs identified in the initial case–control study could not be assessed in the incident studies as the methylation arrays differed in the number of CpGs (EPIC VS 450 k, respectively). Second, we used self-reported information about cardiovascular risk factors in the case–control study, as an event such as MI modifies risk factor levels during the acute phase. Third, we cannot infer causality since changes in methylation could have occurred as a consequence of the acute phase and disease management of the MI event. We aimed to perform MR studies of the association between the identified CpGs and cardiovascular events, but available methylation Quantitative Trait Loci (meQTL) datasets are still limited. Last, our study is based on populations of European origin and the results cannot be extrapolated to other populations.

## Conclusion

Our study provides 34 novel DNA methylation loci related to MI. The results shed some light on the molecular landscape of MI, highlighting the importance of traditional CVRFs and inflammation in the development of CHD. Our results question the relevance of DNA methylation as a predictive biomarker.

## Methods

### Study design and populations

We designed an EWAS using three populations: the Girona Heart Registry (REGICOR, REgistre GIroní del COR), the Women’s Health Initiative (WHI), and the Framingham Offspring Study (FOS). We first performed a two-stage EWAS on acute MI using two independent age- and sex-matched case–control studies designed in REGICOR. Then, we validated the results in the other two populations with incident cases of CHD and CVD.

#### Case–control studies of acute MI in REGICOR

The sample used in the discovery stage (REGICOR-1) involved 416 individuals (208 MI cases and 208 controls). The sample in the validation stage (REGICOR-2) comprised 208 individuals (104 cases and 104 controls). Cases were selected from patients who were consecutively attended for a first acute MI in the reference hospital of the monitored area, in the province of Girona, in the northeast of Spain. Women were overrepresented to achieve their inclusion as 50% of our sample. Controls were participants in a population-based survey performed in the same monitored area. They were randomly selected from those attending the 2009–2013 follow-up visit (n = 4980), and matched by age and sex with the MI cases. All participants were of European descent and provided informed written consent. The study was approved by the local ethics committee (2015/6199/I; 2018/7855/I) and meets the principles expressed in the Declaration of Helsinki and the relevant Spanish legislation.

#### Samples with incident cases of CHD and CVD

The WHI sample is a case–control study nested in a cohort. The FOS sample is a prospective cohort study. Both samples were available in the database of Genotypes and Phenotypes (http://dbgap.ncbi.nlm.nih.gov; Project Number #9047). The graphical abstract shows the design and flow-chart of this study.

### Assessment of cardiovascular outcomes

The outcomes assessed were acute MI in REGICOR, and incident CHD and CVD in the WHI and FOS samples. Additional details are provided in the Additional file 1: Methods.

### Assessment of DNA methylation

DNA methylation was assessed genome-wide from peripheral blood with commercial arrays from Illumina (CA, USA). The Infinium MethylationEPIC BeadChip, covering over 850,000 CpGs, was used in the REGICOR samples. The Infinium HumanMethylation450 BeadChip, covering over 480,000 CpGs, was used in the WHI and FOS samples. A detailed quality control pipeline for the methylation data is available in the Additional file 1: Methods. Methylation status at each CpG was reported by β-values [30].

### Covariates

In the REGICOR case–control studies the following covariates were considered: self-reported smoking, diabetes, hypercholesterolemia and hypertension (Additional file 1: Methods). In the WHI and FOS studies self-reported smoking and glycaemia, total and HDL cholesterol, and blood pressure measurements were considered. Moreover, we inferred the peripheral blood cell counts with the *FlowSorted.Blood.450 k* R package [31]. We also estimated two surrogate variables for unknown sources of potential technical or biological confounding using the *sva* R package [32].

### Statistical analysis

All statistical analyses were performed using R version 3.4.0. The codes of the Singularity images used to run the EWASs in the high performance computing system of the Hospital del Mar Medical Research Institute are available in the repositories at https://github.com/regicor/methylation_ami/. A detailed description of the statistical methods is provided in the Additional file 1: Methods.

#### Association between DNA methylation and cardiovascular outcomes

Logistic regression was used in the analyses in the REGICOR and WHI samples, while Cox regression was used in the FOS sample. We considered the cardiovascular event (acute MI, CHD or CVD) as the outcome and DNA methylation as the exposure.

We defined three models. Model 1 was adjusted for estimated cell counts and two surrogate variables (plus age and ethnicity in the WHI sample, plus age and sex in the FOS samples). Model 2 was additionally adjusted for smoking. Model 3 was further adjusted for diabetes, hypercholesterolemia and hypertension.

In order to reduce epigenomic inflation, we corrected the coefficients, the standard errors and the *p* values using the *bacon* R package if necessary [33]. The *bacon* R package controls for bias and inflation using a Bayesian method based on the estimation of the empirical null distribution and was used in previous EWAS [33–35]. We used coefficients and standard errors from the regression models as the input data and we set a random seed at 123.

We selected those associations from the discovery stage (REGICOR-1) with a corrected *p-value* < 10^–5^ for assessment in the validation stage (REGICOR-2). Moreover, we performed a fixed-effect meta-analysis of the corrected effect sizes observed in both stages, weighted by the inverse of the variance. Thereafter, we studied the association of the identified CpGs with incident CHD and with CVD events in the WHI and the FOS samples, separately. The results from both samples were meta-analysed (for CHD and CVD, separately). We used the Bonferroni criteria to correct for multiple comparisons (0.05 divided by the number of probes analysed in each specific analysis).

#### Association between the identified CpGs and CVRFs

We analysed whether the methylation levels of the identified CpGs were associated with individual CVRFs in the four samples using multiple linear regression, and then meta-analysed the results. We defined DNA methylation as the outcome and adjusted for age and sex in the case of the REGICOR and the Framingham populations, and for age and ethnicity in the WHI sample. In the case of the REGICOR samples, the continuous variables were only available for the control individuals. We meta-analysed the results from the four populations using a fixed-effects meta-analysis weighted by the inverse of the variance. The *p* value threshold was estimated as 0.05 divided by the multiplication of the number of CVRFs and the number of CpGs assessed.

#### Methylation risk scores (MRSs) and predictive capacity

We developed two weighted MRSs based on the CpGs identified, each of them using the results from the meta-analyses of incident CHD or CVD, respectively. We evaluated the association between these scores and CHD and CVD incidence, respectively, in the FOS sample, using Cox regression. All analyses were adjusted for age, sex, diabetes, smoking, systolic blood pressure, hypertensive treatment, and levels of total cholesterol and HDL-C [36]. We also assessed the potential added predictive value of including the MRSs in the Framingham risk function. We evaluated the increase in the discrimination and the reclassification.

### Causality of associations between DNA methylation and cardiovascular outcomes

We took a two-sample Mendelian Randomization studies using the MR-Base platform [37]. We used the MRInstruments R package to select the instrumental variables, and then, the TwoSampleMR R package. First, we considered those methylation-level quantitative trait loci (meQTL) from the Accesible Resource for Integrated Epigenomic Studies (ARIES) project [16] included in the MR-Base database [37]. Then, we interrogated their association with MI and with CHD using summary statistic data from a meta-analysis of GWAS on CHD [38]. A more detailed description of the analysis is included in the Additional file 1: Methods.

## Supplementary Information

**Additional file 1.** Additional material.

**Additional file 2: Table S1.** Discovery stage of the EWAS on acute myocardial infarction (REGICOR-1 sample). Model 1 was adjusted for estimated cell counts and two surrogate variables. Model 2 was further adjusted for smoking status. Model 3 was additionally adjusted for diabetes, hypercholesterolemia and hypertension. Coefficients, standard errors and p-values are given for each model before and after the correction of the inflation using the bacon R package. Suggestive significant associations (p-value<10-5) are in bold. The total number of suggestive significant associations in each model is given. **Table S2.** Meta-analyses of the results from the discovery (REGICOR-1) and the validation stage (REGICOR-2). Model 1 was adjusted for estimated cell counts and two surrogate variables. Model 2 was further adjusted for smoking status. Model 3 was additionally adjusted for diabetes, hypercholesterolemia and hypertension. Coefficients, standard errors and p-values of REGICOR-1 are those corrected with the bacon R package. Significant associations (p-value<6.17 × 10-8) are highlighted in bold. The total number of significant associations in each model is given. **Table S3.** Meta-analysis of the results from the follow-up association studies performed in the samples with incident cases of cardiovascular (CVD) and coronary heart disease (CHD). Model 1 was adjusted for age, estimated cell counts and two surrogate variables (plus ethnicity in WHI and sex in FOS). Model 2 was further adjusted for smoking status. Model 3 was additionally adjusted for diabetes, hypercholesterolaemia and hypertension. Significant associations (4.17 × 10-3) found in the fixed-effects meta-analysis are highlighted in bold. The total number of significant associations in each model is given. **Table S4.** Utility of the methylation risk scores (MRS): association with cardiovascular (CVD) or coronary (CHD) incidence and assessment of their predictive capacity. MRSs were based on the results from model 1 of incident CHD and CVD. Analyses of the association with the CVD or CHD incidence were adjusted for age, sex, total cholesterol and HDL-C levels, diabetes, smoking status, systolic blood pressure, and hypertensive treatment, which are the cardiovascular risk factors considered in the Framingham risk function. Analysis of the improvement in the predictive capacity of the Framingham function was performed with and without the corresponding MRS. Table S5. Results of the Wald ratio method applied to determine the causality between the identified CpGs and coronary heart disease or myocardial infarction.

## Data Availability

All data generated during this study are included in this published article and its Additional files. The REGICOR datasets analysed during the current study are available from the corresponding author on reasonable request.

## Abbreviations

450 k: Infinium HumanMethylation450 BeadChip
MI: Myocardial infarction
CHD: Coronary heart disease
CVD: Cardiovascular disease
CVR: Cardiovascular risk
CVRF: Cardiovascular risk factor
EPIC: Infinium MethylationEPIC BeadChip
EWAS: Epigenome-wide association study
FOS: Framingham offspring study
MRS: Methylation risk score
REGICOR: REgistre GIroní del COR
WHI: Women’s Health Iniciative
