# Supplementary material for: DNA methylation biomarkers of myocardial infarction and cardiovascular disease
Source: Clin Epigenetics. 2021 Apr 21;13:86. doi: 10.1186/s13148-021-01078-6 (PMC8061080; doi:10.1186/s13148-021-01078-6)
Supplement: Supplementary file 1 — Additional file 1. Additional material. [file 13148_2021_1078_MOESM1_ESM.docx]

**ADDITIONAL MATERIAL**

**ADDITIONAL METHODS**

**Assessment of cardiovascular outcomes**

*a.-* *REGICOR:* Acute myocardial infarction (AMI) was defined according to international World Health Organization criteria^1^ when two or more of the following were present: abnormal new Q waves, increase in cardiac enzymes beyond twice the upper normal value, and chest pain lasting more than 20 min.

*b.-* *WHI*: coronary heart disease (CHD) was considered when the participant had experienced any of the following: clinical AMI, definite silent AMI, total AMI, angina, coronary revascularization procedure or CHD death. Cardiovascular disease (CVD) included CHD and stroke or death due to cerebrovascular disease or other CVD. Clinical events were adjudicated in 2016 using the form 121, which considered events requiring hospitalization or occurring during a hospitalization for another reason. Diagnosis of a stroke was completed by a stroke neurologist in form 132. The cause of death was recorded using form 124.

c.- *FOS*: data regarding CHD and CVD were extracted from follow-up for cardiovascular events through 2014 (until exam 12). We used exam 8 as the baseline visit and to identify prevalent cases.

**Assessment of DNA methylation status**

In the REGICOR study, DNA was extracted from whole peripheral blood drawn within the first 24 hours post-AMI for cases and at follow-up for controls. For the other samples, DNA was extracted from buffy coat obtained from whole peripheral blood samples collected at baseline in the WHI population^2^ and at exam 8 in the FOS.^3^

DNA methylation was assessed genome-wide with commercial arrays based on bisulfite conversion of unmethylated cytosines. In the REGICOR study, we used the Infinium MethylationEPIC BeadChip (Illumina, CA, USA). This array analyses over 850,000 CpGs per sample.^4^ After checking the DNA quality with Picogreen (Thermo Fisher Scientific, MA, USA), samples of REGICOR-1 were analysed in 13 batches of the Infinium MethylationEPIC BeadChip in the Genomics and Epigenomics Service of the Bellvitge Institute for Biomedical Research (Barcelona, Spain). Samples of REGICOR-2 were also analyzed at the same facilities, in three batches. In the WHI and FOS studies, the Infinium HumanMethylation450 BeadChip (Illumina, CA, USA) was used. This array analyses over 485,000 CpGs per sample,^5^ from which 439,562 are included in EPIC BeadChip.^4^ Assessment of DNA methylation for these two samples has been described elsewhere.^2,6^

**Quality control of DNA methylation data**

Quality control of the raw methylation data was previously described for the Infinium HumanMethylation450 BeadChip data by our group.^7^ This pipeline was applied to FOS and WHI.

*Quality control of the samples analysed with the Illumina MethylationEPIC BeadChip*

We removed the samples with a detection p-value >0.05 in at least 1% of the probes using the *pfilter* function of the *wateRmelon* R package available through the Bioconductor repository. We also discarded those samples that did not cluster in the corresponding sex cluster based on the DNA methylation levels in the X chromosome using the *methylumi* R package available through the Bioconductor repository.

*Quality control of the CpGs analysed with the Illumina MethylationEPIC BeadChip*

We excluded those probes with both a detection p-value >0.05 in at least 1% of the samples and a beadcount <3 in at least 5% of the samples using *wateRmelon* R package available through the Bioconductor repository. We further removed those probes reported by Illumina to be discarded due to underperformance (n=1,031) and changes in the manufacturing process (n=977). Finally, we excluded those probes corresponding to a methylation site different from a CpG site and those that could hybridize in more than one genomic region (n=43,979).^8,9^

**DNA methylation measurement**

Methylation status at each CpG site was reported by β-values, which are more intuitively interpreted than M-values.^10^ β-values range between 0 (completely unmethylated) and 1 (completely methylated).

They were calculated according to equation 1:

$\beta-value=\frac{M_{i}}{M_{i}+U_{i}+\alpha}$ Equation 1

Where:

- M_i_ = intensity of methylated probe,

- U_i_ = intensity of unmethylated probe, and

- α = 100; constant offset.

To remove potential sources of technical variation not related to the underlying biology, we standardized the β-values by batch, as described previously by our group,^7^ using Equation 2:

$Z=\frac{(X-\bar{X})}{\sqrt{\frac{\sum{(X-\bar{X})}^{2}}{(n-1)}}}$ Equation 2

Where:

- Z = standardized β-value,

- X = β-value for a specific individual,

- X̄ = mean of β-value for a specific batch, and

- n = sample size.

Finally, to avoid the influence of extreme values, we excluded those CpGs with a β-value 4 standard deviations higher and lower from the mean.

**Genomic information**

We obtained genomic information of the CpGs using the manifest and annotation provided by Illumina and contained in the corresponding R packages available through the Bioconductor repository (*IlluminaHumanMethylation450kanno.ilmn12.hg19* and *IlluminaHumanMethylationEPICanno.ilm10b2.hg19*).

**Covariates assessment**

The REGICOR study specifically trained a group of nurses to collect blood samples and sociodemographic, lifestyle, and cardiovascular risk factors information using validated methods and questionnaires.^11^ Data from the FOS and the WHI sample were obtained through the Genotypes and Phenotypes database (http://dbgap.ncbi.nlm.nih.gov; project number #9047). The procedures used to collect data and blood samples of the REGICOR, WHI and the FOS populations were previously described.^2,12,13^ We considered the following covariates:

- *Smoking*: self-reported and categorized as current smokers (at least 1 cigarette/day or quitted smoking within the year before the visit) or non-smokers (never smoked or quit smoking at least one year before the visit),
- *Diabetes*: self-reported diabetes or treatment (REGICOR), glucose levels ≥126 mg/dL or treatment (WHI and FOS),
- *Hypercholesterolemia*: self-reported high cholesterol levels or treatment (REGICOR), total cholesterol ≥240 mg/dL or LDL-C ≥160 mg/dL or treatment (WHI and FOS),
- *Hypertension*: self-reported hypertension or treatment (REGICOR), SBP ≥140 mmHg or DBP ≥90 mmHg or treatment (WHI and FOS),
- *Estimated peripheral blood cell counts*,^14^
- *Two surrogate variables*.^15^

When applicable, we excluded from analysis those individuals with no information available regarding the cardiovascular risk factors.

**Statistical analysis**

*Methylation risk score (MRS)*

The MRSs summarize an individual’s epigenetic predisposition to suffer a cardiovascular event. The weights for each CpG were based on the coefficients of the meta-analysis of Model 1 following Equation 3:

$MRS=\sum_{i=1}^{N} \beta_{i}^{meta}\cdot\beta_{i}^{meth}$ Equation 3

Where:

- MRS = methylation risk score for a specific individual,

- i = CpG,

- N = CpG sample size,

- β_meta_ = coefficient of the meta-analysis for each CpG, and

- β_meth_ = standardized β-value for each CpG.

*Evaluation of the predictive capacity of CVR functions including the MRSs*

The estimated risk for each individual was computed using Cox regression, according to Equation 4:

$CVR=1-S_{\bar{X}}^{\sum_{j=1}^{p} \beta_{j}^{F}\cdot(F_{j}-\bar{F}_{j})+\beta^{MRS}\cdot(MRS-\bar{MRS})}$ Equation 4

Where:

- *1-S* = probability of presenting a CVD event in the next 10 years based on the incidence of CVD in the population,

- *β^F^* = effect size of each CVRF,

- *F_j_* = CVRFs per individual (logarithm of age, total cholesterol and HDL-C, and SBP (treated and not treated), sex, smoking status, hypertension treatment and diabetes),

- $\bar{F}_{j}$= population mean of the CVRFs (logarithm of age, total cholesterol and HDL-C, and SBP (treated and not treated), sex, smoking status, hypertension treatment and diabetes),

- *β^MRS^* = effect size of the MRS,

- *MRS* = MRS per individual, and

- $\bar{MRS}$= population mean of the CVRFs.

First, we evaluated the calibration of the models using the Hosmer-Lemeshow test.^16^ Next, we assessed the discriminative capacity of the models using the concordance index (c-statistic),^17^ applying the *rcorr.cens* function of the *Hmisc* R package. Last, we calculated the reclassification improvement using the net reclassification improvement (NRI) index^18^ with the *nricens* R package. In this regard, we defined three risk categories (low, intermediate and high) with cut-off points defined according to guidelines for 10-year risk reported by the NCEP Panel:^19^ [0–10)%, [10–20)%, ≥20%, respectively). We calculated the expected number of events at 5 years in each risk category (thus, [0–5)%, [5–10)%, ≥10%) using Kaplan-Meier estimates. Confidence intervals for the Kaplan-Meier estimates were obtained from the bootstrapping method applied by the *nricens* R function (*niter* = 1,000 bootstrap samples). We also analysed the NRI in the group of individuals with intermediate CVR (clinical NRI). To correct for bias in the NRI estimation among individuals with intermediate risk, we used the method proposed by Paynter and Cook.^20^

**Analysis of the causality of associations between DNA methylation and cardiovascular outcomes**

To run the mendelian randomization analyses, we selected the following methods as options: (a) clumping to prune SNPs for linkage disequilibrium (LD); (b) proxy SNPs through LD tagging (minimum LD R^2^=0.8) if one SNP is not present in an outcome dataset, allowing palindromic SNPs (MAF threshold for alignment=0.3); (c) alignment of strands for palindromic SNPs for allele harmonization (i.e. effects of the SNPs on DNA methylation and on the outcome correspond to the same allele); and (d) Wald ratio, maximum likelihood, MR Egger, weighted median, Inverse variance weighted, Inverse variance weighted (fixed effects), and weighted mode.

**ADDITIONAL REFERENCES**

1. Gillum RF, Fortmann SP, Prineas RJ, Kottke TE. International diagnostic criteria for acute myocardial infarction and acute stroke. *American Heart Journal*. 1984;108(1):15-8.
2. Levine ME, Hosgood HD, Chen B, Absher D, Assimes T, Horvath S. DNA methylation age of blood predicts future onset of lung cancer in the women’s health initiative. *Aging*. 2015;7(9):690-700.
3. Irvin MR, Zhi D, Joehanes R, Mendelson M, Aslibekyan S, Claas SA, Thibeault KS, Patel N, Day K, Jones LW, Liang L, Chen BH, Yao C, Tiwari HK, Ordovas JM, et al. Epigenome-Wide Association Study of Fasting Blood Lipids in the Genetics of Lipid-Lowering Drugs and Diet Network Study. *Circulation*. 2014;130(7):565-72.
4. Moran S, Arribas C, Esteller M. Validation of a DNA methylation microarray for 850,000 CpG sites of the human genome enriched in enhancer sequences. *Epigenomics*. 2016;8(3):389-99.
5. Sandoval J, Heyn H, Moran S, Serra-Musach J, Pujana MA, Bibikova M, Esteller M. Validation of a DNA methylation microarray for 450,000 CpG sites in the human genome. *Epigenetics*. 2011;6(6):692-702.
6. Irvin MR, Zhi D, Joehanes R, Mendelson M, Aslibekyan S, Claas SA, Thibeault KS, Patel N, Day K, Jones LW, Liang L, Chen BH, Yao C, Tiwari HK, Ordovas JM, et al. Epigenome-Wide Association Study of Fasting Blood Lipids in the Genetics of Lipid-Lowering Drugs and Diet Network Study. *Circulation*. 2014;130(7):565-72.
7. Sayols-Baixeras S, Lluís-Ganella C, Subirana I, Salas LA, Vilahur N, Corella D, Muñoz D, Segura A, Jimenez-Conde J, Moran S, Soriano-Tárraga C, Roquer J, Lopez-Farré A, Marrugat J, Fitó M, et al. Identification of a new locus and validation of previously reported loci showing differential methylation associated with smoking. The REGICOR study. *Epigenetics*. 2015;10(12):1156-65.
8. Pidsley R, Zotenko E, Peters TJ, Lawrence MG, Risbridger GP, Molloy P, Van Djik S, Muhlhausler B, Stirzaker C, Clark SJ. Critical evaluation of the Illumina MethylationEPIC BeadChip microarray for whole-genome DNA methylation profiling. *Genome biology*. 2016;17(1):208.
9. McCartney DL, Walker RM, Morris SW, Mcintosh AM, Porteous DJ, Evans KL. Identification of polymorphic and off-target probe binding sites on the Illumina Infinium MethylationEPIC BeadChip. *Genom Data*. 2016;9:22-4.
10. Du P, Zhang X, Huang C-C, Jafari N, Kibbe WA, Hou L, Lin SM. Comparison of Beta-value and M-value methods for quantifying methylation levels by microarray analysis. *BMC Bioinformatics*. 2010;11(1):587.
11. Grau M, Subirana I, Elosua R, Solanas P, Ramos R, Masia R, Cordón F, Sala J, Juvinyà D, Cerezo C, Fitó M, Vila J, Covas MI, Marrugat J. Trends in cardiovascular risk factor prevalence (1995-2000-2005) in northeastern Spain. *European Journal of Preventive Cardiology*. 2007;14(5):653-9.
12. Sentí M, Tomás M, Marrugat J, Elosua R, REGICOR Investigators. Paraoxonase1-192 polymorphism modulates the nonfatal myocardial infarction risk associated with decreased HDLs. *Arteriosclerosis, thrombosis, and vascular biology*. 2001;21(3):415-20.
13. Sayols-Baixeras S, Subirana I, Lluis-Ganella C, Civeira F, Roquer J, Do A, Absher D, Cenarro A, Muñoz D, Soriano-Tárraga C, Jiménez-Conde J, Ordovas JM, Senti M, Aslibekyan S, Marrugat J, et al. Identification and validation of seven new loci showing differential DNA methylation related to serum lipid profile: an epigenome-wide approach. The REGICOR study. *Human Molecular Genetics*. 2016;25(20):ddw285.
14. Houseman EA, Accomando WP, Koestler DC, Christensen BC, Marsit CJ, Nelson HH, Wiencke JK, Kelsey KT. DNA methylation arrays as surrogate measures of cell mixture distribution. *BMC bioinformatics*. 2012;13(1):86.
15. Leek JT, Johnson WE, Parker HS, Jaffe AE, Storey JD. The sva package for removing batch effects and other unwanted variation in high-throughput experiments. *Bioinformatics*. 2012;28(6):882-3.
16. D’Agostino RB, Nam B-H. Evaluation of the Performance of Survival Analysis Models: Discrimination and Calibration Measures. *Handbook of Statistics*. 2003;23:1-25.
17. Newson R. Confidence Intervals for Rank Statistics: Somers’ D and Extensions. *The Stata Journal: Promoting communications on statistics and Stata*. 2006;6(3):309-34.
18. Pencina MJ, D’Agostino RB, Steyerberg EW. Extensions of net reclassification improvement calculations to measure usefulness of new biomarkers. *Statistics in Medicine*. 2011;30(1):11-21.
19. National Cholesterol Education Program (NCEP) Expert Panel on Detection, Evaluation, and Treatment of High Blood Cholesterol in Adults (Adult Treatment Panel III). Third Report of the National Cholesterol Education Program (NCEP) Expert Panel on Detection, Evaluation, and Treatment of High Blood Cholesterol in Adults (Adult Treatment Panel III) final report. *Circulation*. 2002;106(25):3143-421.
20. Paynter NP, Cook NR. A bias-corrected net reclassification improvement for clinical subgroups. *Medical decision making: an international journal of the Society for Medical Decision Making*. 2013;33(2):154-62.

**ADDITIONAL FIGURES**

**Additional Figure 1.** Manhattan plots of the associations between DNA methylation and acute myocardial infarction in the discovery stage (REGICOR-1). Model 1 was adjusted for estimated cell counts and two surrogate variables. Model 2 was further adjusted for smoking status. Model 3 was additionally adjusted for diabetes, hyperlipidaemia and hypertension. Inflation was corrected using the bacon R package; plots are given before (left) and after (right) the correction.

| **Model 1** | **Non-corrected** | **Corrected** |
| --- | --- | --- |
|  | **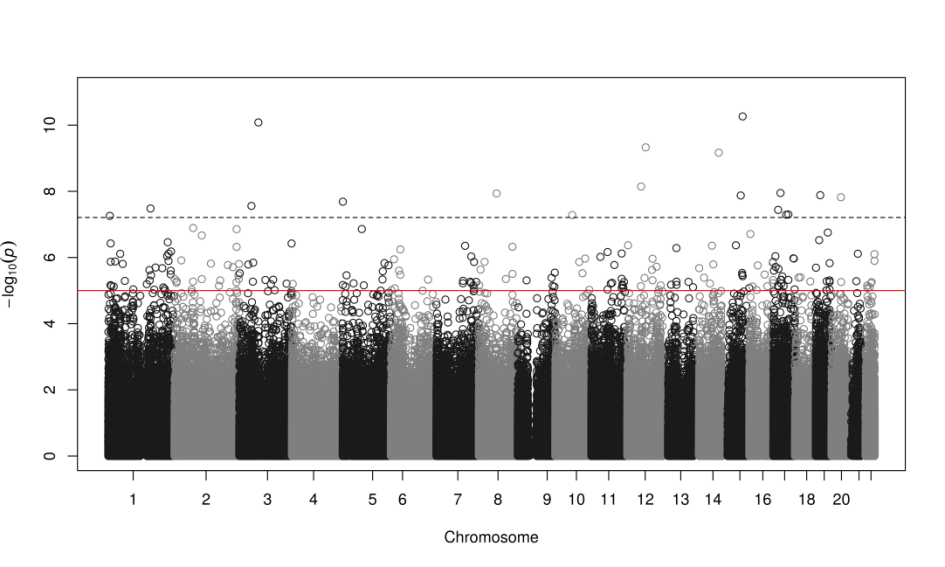** | **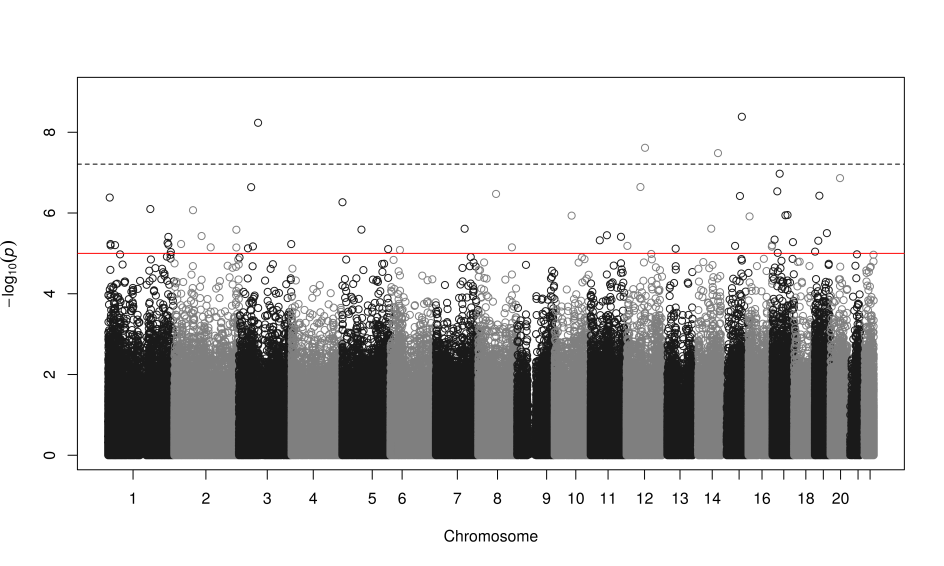** |

|  | **Non-corrected** | **Corrected** |
| --- | --- | --- |
| **Model 2** | **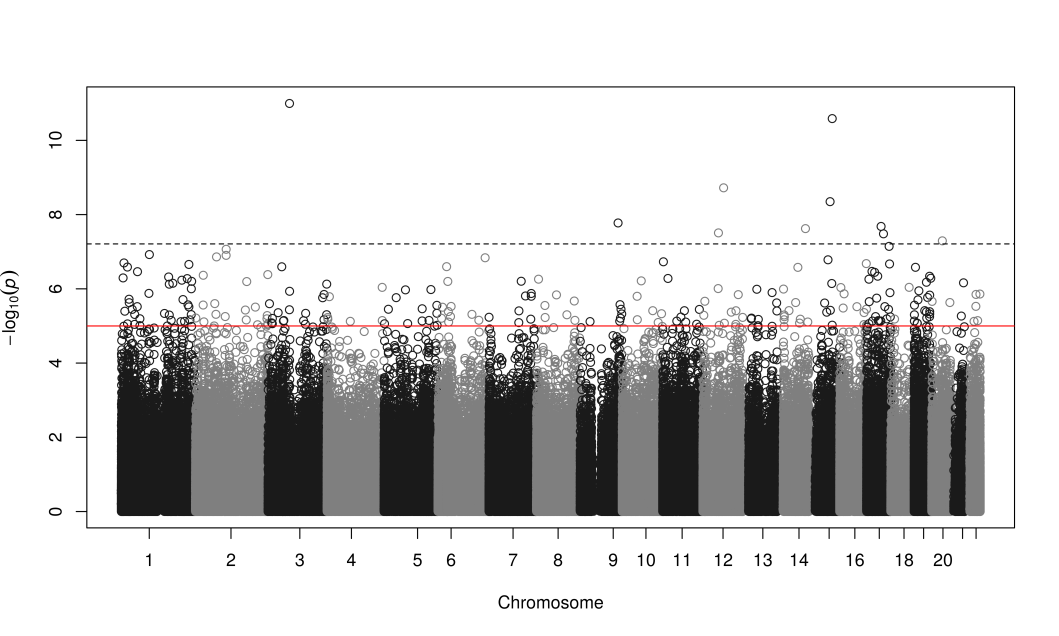** | **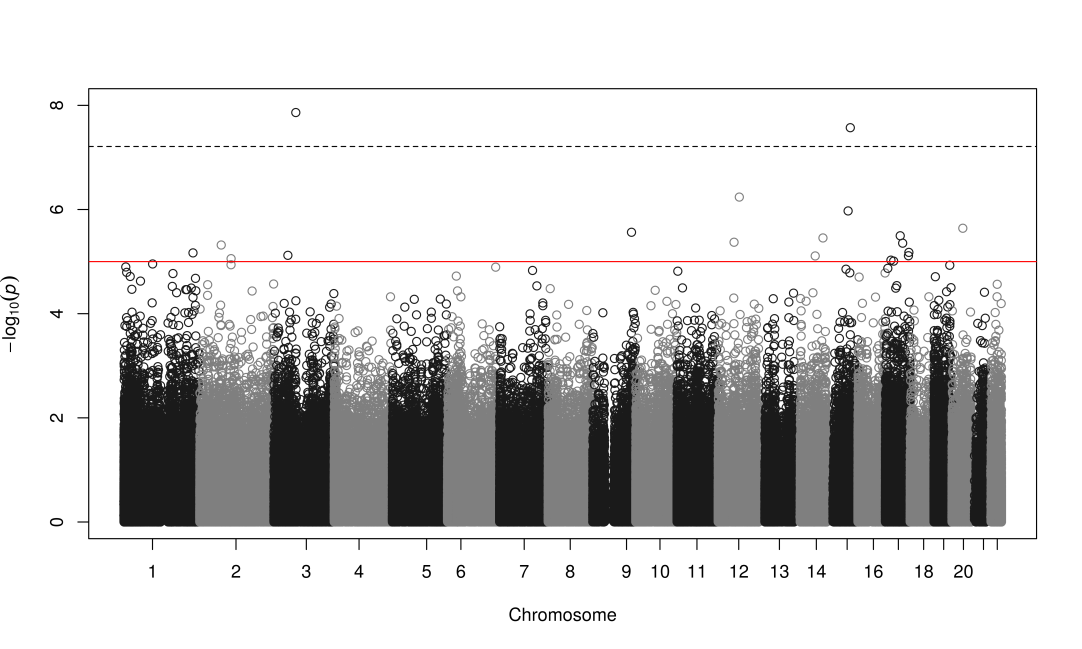** |
|  | **Non-corrected** | **Corrected** |
| **Model 3** | **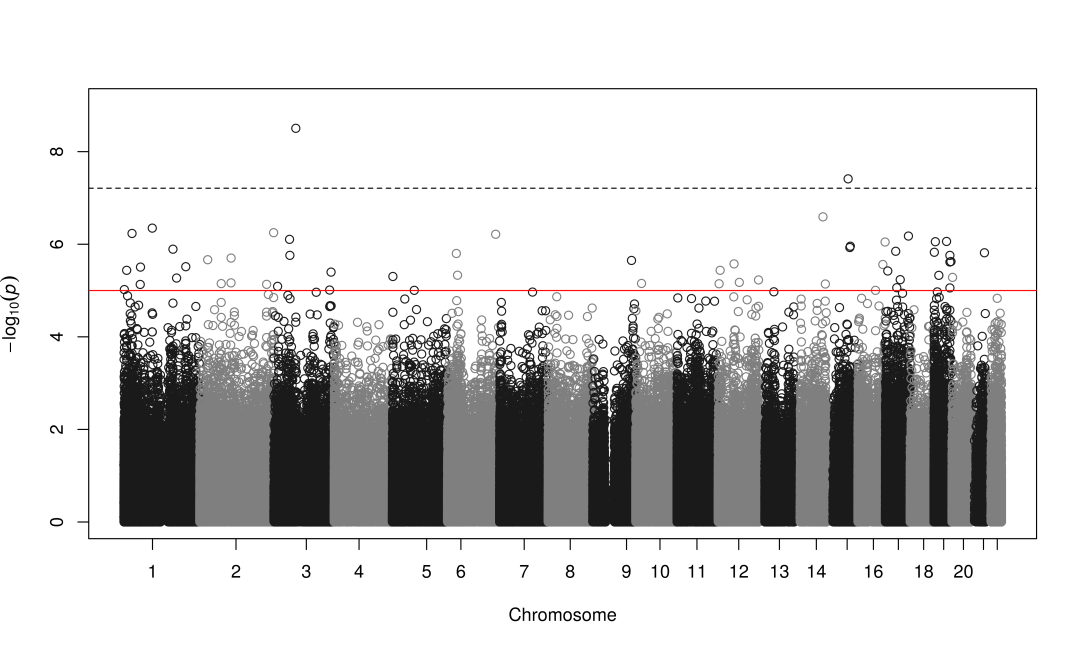** | **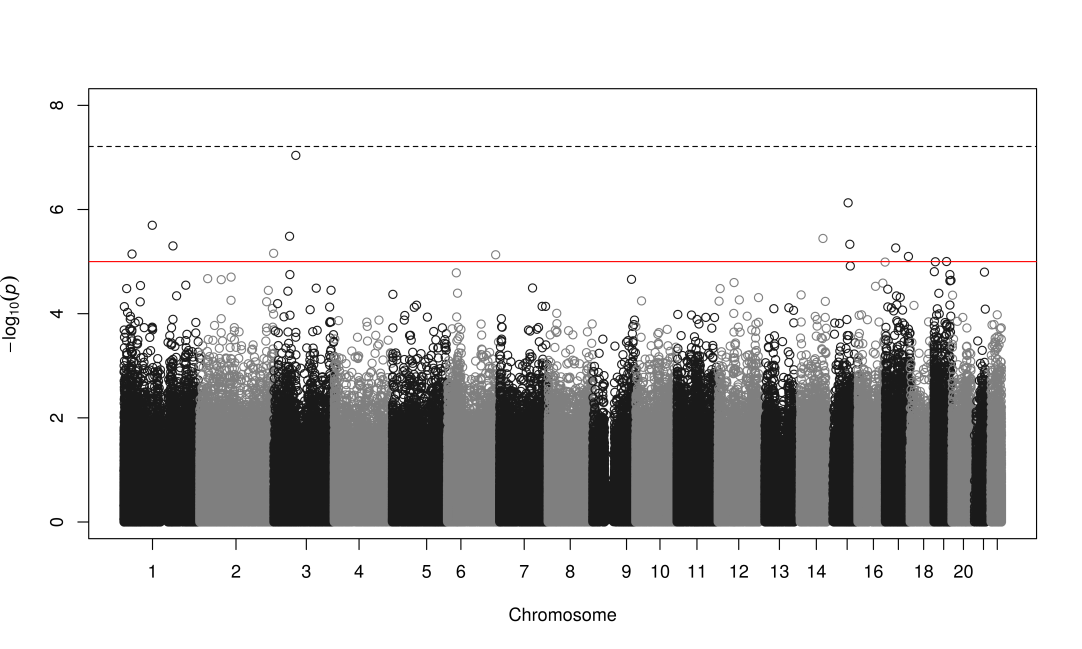** |

**Additional Figure 2.** QQ plots of the associations between DNA methylation and acute myocardial infarction in the discovery stage (REGICOR-1). Model 1 was adjusted for estimated cell counts and two surrogate variables. Model 2 was further adjusted for smoking status. Model 3 was additionally adjusted for diabetes, hyperlipidaemia and hypertension. Inflation was corrected using the bacon R package; plots are given before (left) and after (right) the correction.

|  | **Model 1** | **Model 2** | **Model 3** |
| --- | --- | --- | --- |
| **Non-corrected** | **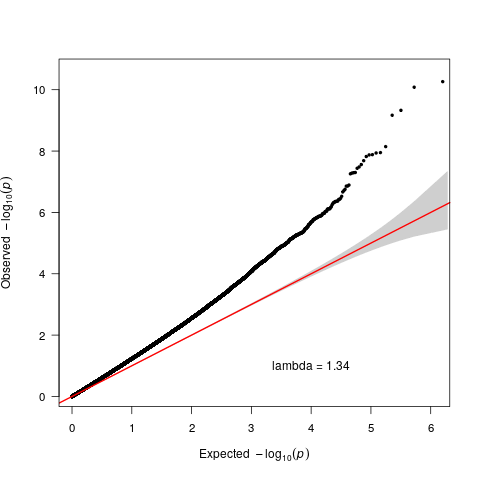** | **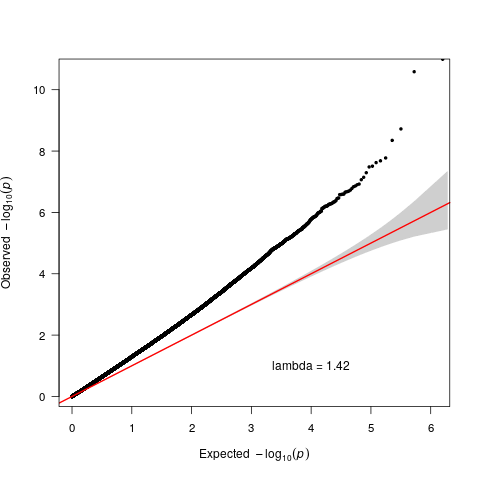** | **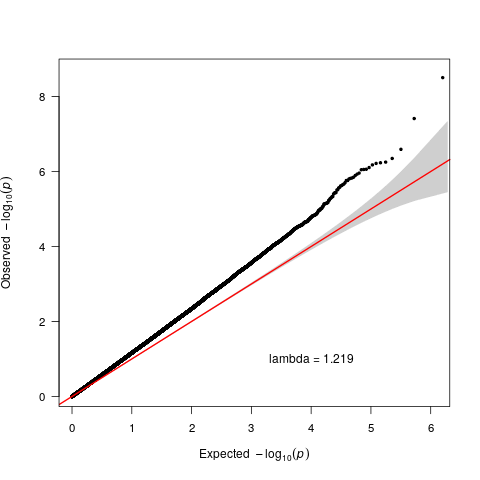** |
| **Corrected** | **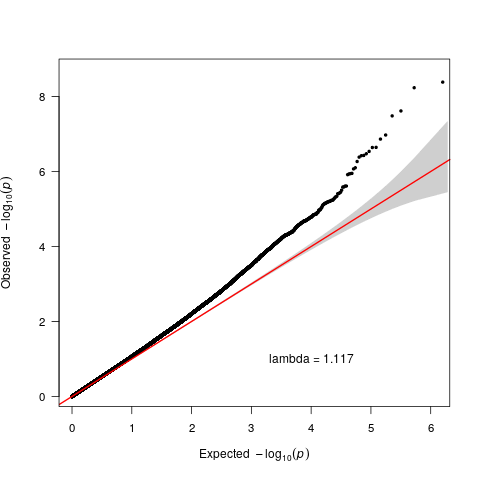** | **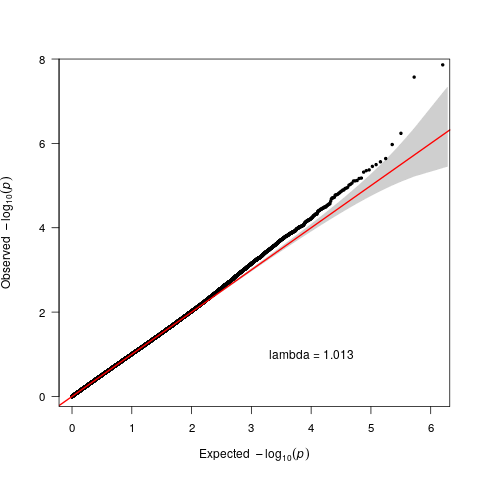** | **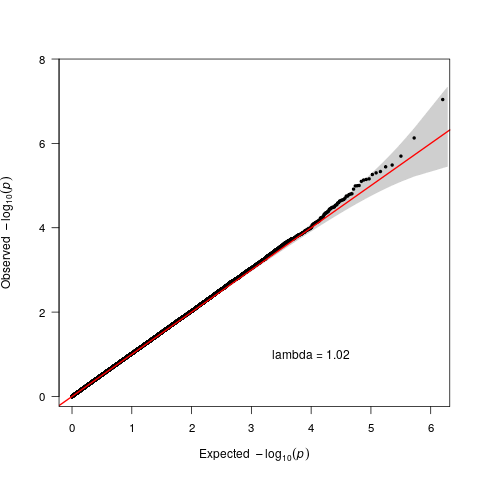** |

**Additional Figure 3. Venn diagram showing the overlap of CpGs found in the three models.** The number of CpGs instead of their identifiers is given.

**
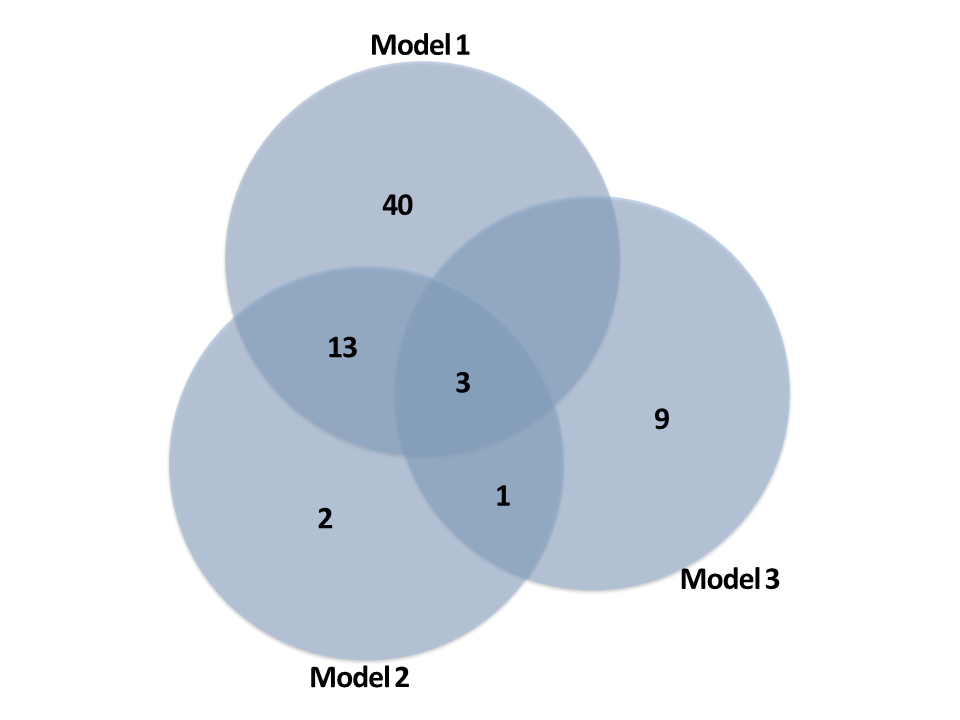
**
